# Discoveries in the pathophysiology of neuropsychiatric lupus erythematosus: consequences for therapy

**DOI:** 10.1186/1741-7015-11-91

**Published:** 2013-04-04

**Authors:** Takahisa Gono, Yasushi Kawaguchi, Hisashi Yamanaka

**Affiliations:** 1 Institute of Rheumatology, Tokyo Women’s Medical University, 10-22 Kawada-cho, Shinjuku-ku, Tokyo, 162-0054, Japan

**Keywords:** Systemic lupus erythematosus, Autoantibody, Cross-reactivity, Neuropsychiatric symptoms

## Abstract

Systemic lupus erythematosus (SLE) is a multi-system inflammatory disorder characterized by the presence of several autoantibodies, including anti-double-stranded DNA. Neuropsychiatric (NP)LE contributes to the prognosis of SLE, and is divided into 19 NPLE syndromes. Its mechanisms are mediated through autoantibodies, complement components, and cytokines. The pathophysiology and diagnosis of NPLE are diverse and complicated. Recent studies have shown that several autoantibodies cross-react with human brain tissue and cause NPLE symptoms in SLE. It is known that in mice, depression and hippocampus-related memory impairment are induced by anti-ribosomal P antibody and anti-NR2 antibody, respectively. In a *BMC Medicine* research article, Kivity et al. demonstrated novel work showed that the 16/6 Id antibody impaired visual memory and spatial memory by causing hippocampal injury in mice. Given differences in the cross-reactivity of each autoantibody with the nervous system, the clinical features might be different and diverse in NPLE. Identification of autoantibody targets could lead to the development of novel therapies. Investigators and clinicians should consider not only the inhibition of autoantibody synthesis but also the protection of neuronal cells in the treatment strategy for NPLE.

See related Research article: http://www.biomedcentral.com/1741-7015/11/90

## Introduction

Systemic lupus erythematosus (SLE) is a multi-system inflammatory disorder characterized by the presence of autoantibodies directed against double-stranded (ds) DNA. The prevalence ranges from 20 to 150 cases per 100,000 population, and it seems to be increasing, partly because the disease is recognized more readily and partly because of longer survival [1]. Specifically, lupus nephritis, which is a kidney disorder that is a complication of SLE, and neuropsychiatric (NP)LE contribute to the prognosis of SLE.

NPLE is classified by the American College of Rheumatology (ACR) into 19 neuropsychiatric syndromes [2]. The diffuse central nervous system (CNS) form, focal CNS form, and peripheral nervous system (PNS) form were defined as diffuse psychiatric/neuropsychological syndromes, neurologic syndromes, and PNS syndromes, respectively, by the ACR. The pathophysiology of NPLE is mediated by several factors, including vasculitis, thromboembolism, and inflammation and apoptosis of neuronal and glial cells. Its mechanisms are mediated through autoantibodies, complement components, cytokines, chemical mediators, and inflammatory cells, including neutrophils, lymphocytes, and plasma cells. Thus, the pathophysiology and diagnosis of NPLE are diverse and complicated, which makes therapy difficult. The diagnosis of NPLE is based on the results of several investigations, including neurological examination, brain/spinal cord magnetic resonance imaging, electroencephalography, cerebrospinal fluid analysis, nerve-conduction studies, psychiatric interview, and a short battery of neuropsychological tests recommended by the ACR committee [2].

Recent novel studies have identified some aspects of the pathophysiology of NPLE. Some anti-dsDNA antibodies cross-react with the *N*-methyl-D-aspartate (NMDA) receptor subunit 2 (NR2) in SLE [3]. NMDA receptors are ligand-gated ion channels that play crucial roles in synaptic transmission and CNS plasticity. NMDA receptor dysfunction has been implicated in multiple brain disorders, including stroke, chronic neurodegeneration, epilepsy, and schizophrenia. Anti-NR2 antibodies breaching the blood–brain barrier (BBB) can cause neuronal damage via an apoptotic pathway [4,5].

The 16/6 idiotype (Id) antibody, which was the focus of a recent study by Kivity et al. [6], is considered to be an anti-dsDNA Id antibody in SLE. Immunization of naive mice with the human 16/6 Id monoclonal antibody induced an SLE-like disease characterized by serological, clinical, and pathological features. This antibody cross-reacts with cytoskeletal proteins, glycoproteins, and brain glycolipids, as well as with pathogens such as *Mycobacterium tuberculosis*[7]. Deposition of 16/6 Id antibodies has been found in human tissues, such as the skin, kidney, and brain [8], and levels are high in patients with active SLE or NPLE [9]. These findings indicate that the 16/6 Id antibody is potentially one of the factors that cause NPLE. However, the way in which these neuropsychiatric symptoms are induced by the 16/6 Id antibody reaching the CNS and the underlying mechanisms of this induction are unknown. In their study, Kivity et al. showed for the first time the effect of the 16/6 Id antibody on the CNS by injecting naive mice intracerebroventricularly with the 16/6 Id antibody [6]. In this commentary, we discuss their results and the pathophysiology and treatment strategy for NPLE.

### Neurological effects of the 16/6 Id antibody

To understand if the 16/6 Id IgG antibody is able to induce neurological effects, Kivity et al. compared the cognitive and behavioral performance of C3H female mice that had been injected with the human 16/6 Id IgG antibody (16/6 Id mice subset) and those injected with a commercial human IgG (control mice subset) [6]. Visual-recognition memory was assessed by using the novel-object recognition test. The authors found that there was a significant preference for attention to the new object compared with the old object by the control mice, but no difference in preference was found between the new and the old objects by the 16/6 Id mice. This result indicates impairment of visual-recognition memory in the 16/6 Id mice. In the Y-maze test, which assesses spatial memory, the 16/6 Id mice were also found to have impairment of spatial memory.

In addition, the brain pathology of these mice was examined to establish the potential mechanism by which the 16/6 Id IgG antibody is able to exert its neurological effects. In the brain tissue, increased microglial activation was seen in the hippocampus and amygdala, but not in the neurocortex or piriform cortex. In addition, the number of astrocytes in the hippocampus was found to be increased. Taken together, these results show that in mice, the 16/6 Id antibody causes impairment of both visual and spatial memory through hippocampal injury, and might selectively cross-react with some antigens in the hippocampus.

### The 16/6 Id antibody is a novel antibody contributing to the pathophysiology of NPLE

Kivity et al. also showed that, in addition to the anti-ribosomal P and anti-NR2 antibodies, another autoantibody, anti-16/6 Id antibody, can cross-react with human brain tissue and cause NPLE symptoms in SLE [6]. Brain tissue-reactive antibodies in NPLE are thought to be synthesized in the CNS or peripheral organs, such as the lymph nodes and bone marrow [10]. Therefore, if NPLE is associated with antibodies reaching the CNS through the BBB, treatments that not only eliminate brain-tissue-reactive antibodies but also protect the integrity of the BBB should be considered.

Therapy of NPLE is currently difficult. Although corticosteroids and immunosuppressive agents, such as cyclophosphamide, are broadly effective for NPLE, the condition is occasionally refractory to these treatments. Moreover, brain-tissue-reactive autoantibodies might cause irreversible neuronal degeneration via apoptosis. For instance, anti-ribosomal P antibody targets a neuronal surface protein, causing calcium influx and apoptosis [11]. These antibodies specifically bind to neurons in the hippocampus, cingulate cortex, and the primary olfactory piriform cortex, and, in mice, resulted in the induction of depression. These results implicate the olfactory and limbic areas in the pathogenesis of depression in SLE [12]. The anti-NR2 antibody also causes neuronal cell apoptosis, impairs the hippocampus-dependent memory function in mice, stimulates NMDA receptor-mediated synaptic responses and excitotoxicity through enhanced mitochondrial permeability [13]. and decreases cell viability through increased Ca^2+^ influx [5]. Kivity et al. showed that the anti-16/6 Id antibody hampers visual-recognition and spatial memory. Their hypothesis about the pathophysiology of anti-16/6 Id antibody-induced brain involvement was that brain inflammation induces modification of neuronal function and neuronal degeneration [6]. The authors also found increases in astrocyte number and microglial activation in the hippocampus of anti-16/6 Id antibody-injected mice. They suggested that increased astrocytes and activated microglial cells were involved in brain inflammation and therefore, an inflammatory process may affect cognitive impairment in mice injected with anti-16/6 Id antibody. By contrast, there was minimal local activation of astrocytes and microglial cells, and no lymphocytic inflammation in the brains of anti-NR2 antibody-injected mice [3].

It is plausible, therefore, that the wide variety of NPLE syndromes might be caused by differences in the recognition of brain tissue by lupus autoantibodies, such as anti-ribosomal P, anti-NR2, and anti-16/6 Id antibodies. Identification and evaluation of such differences would perhaps be useful in developing therapies for NPLE.

### Future directions and conclusions

In the future, it is hoped that new agents will be developed to improve the prognosis for NPLE. The efficacy of such new agents should be determined through their ability to protect neuronal cells, modulate intracellular Ca^2+^, or regulate the deposition of autoantibodies in NPLE. Memantine is a drug used to treat Alzheimer’s disease, which modulates intracellular Ca^2+^ by blocking NMDA receptors. In addition, the DWEYS pentapeptide, which the anti-NR2 antibody recognizes as an antigen, prevents the anti-NR2 antibody from being deposited in tissues and mediating neuronal excitotoxicity in mice [14].

As Kivity et al. have shown, several autoantibodies against brain tissue are involved in NPLE. Given the differences in the cross-reactivity of each autoantibody with the nervous system, this may explain the difference and diversity of the clinical features in NPLE. Investigators and clinicians should consider not only inhibition of autoantibody synthesis but also protection of neuronal cells when investigating treatment strategies for NPLE.

## Abbreviations

ACR: American college of rheumatology; BBB: Blood–brain barrier; CNS: Central nervous system; ds: Double-stranded; NMDA: *N*-methyl-D-aspartate; NPLE: Neuropsychiatric lupus erythematosus; PNS: Peripheral nervous system; SLE: Systemic lupus erythematosus

## Competing interests

The authors declared that they have no competing interest.

## Authors’ contributions

TG drafted the manuscript, and YK and HY read and revised it. All authors have given final approval of the final manuscript to be published.

## Authors’ information

TG is an assistant professor in the Institute of Rheumatology (IOR), Tokyo Women’s Medical University (TWMU), and is interested in the neurological complications associated with connective tissue disease. YK is an associate professor of Medicine and Rheumatology in TWMU. HY is a professor of Medicine and Rheumatology, and a director of the IOR, TWMU. All authors are board-certified members of the Japan College of Rheumatology.

## Pre-publication history

The pre-publication history for this paper can be accessed here:

http://www.biomedcentral.com/1741-7015/11/91/prepub
